# A Nordic survey of the management of palliative care in patients with head and neck cancer

**DOI:** 10.1007/s00405-020-06310-2

**Published:** 2020-09-01

**Authors:** Helena Boëthius, Tiina Saarto, Göran Laurell, Lovisa Farnebo, Antti A. Mäkitie

**Affiliations:** 1 Department of Otorhinolaryngology, Anaesthetics, Operations and Specialty Surgery Center, Region Östergötland, Linköping, Sweden; 2 Department of Biomedical and Clinical Sciences, Linköping University, Region Östergötland, Linköping, Sweden; 3 Department of Palliative Care, Comprehensive Cancer Center, Helsinki University Hospital and University of Helsinki, Helsinki, Finland; 4 Department of Surgical Sciences, ENT, Uppsala University, 75185 Uppsala, Sweden; 5 Department of Otorhinolaryngology, Head and Neck Surgery, Helsinki University Hospital and University of Helsinki, PO Box 263, 00029 HUS, Helsinki, Finland; 6 Research Program in Systems Oncology, Faculty of Medicine, University of Helsinki, Helsinki, Finland; 7 Division of Ear, Nose and Throat Diseases, Department of Clinical Sciences, Intervention and Technology, Karolinska Institutet and Karolinska Hospital, Stockholm, Sweden

**Keywords:** Head and neck cancer, Palliative care, End-of-life care, Palliative guidelines, Best-supportive care, Early integrated palliative care

## Abstract

**Background:**

The five Nordic countries with a population of 27M people form a rather homogenous region in terms of health care. The management of Head and Neck Cancer (HNC) is centralized to the 21 university hospitals in these countries. Our aim was to survey the current status of organization of palliative care for patients with HNC in the Nordic countries as the field is rapidly developing.

**Materials and methods:**

A structured web-based questionnaire was sent to all the Departments of Otorhinolaryngology—Head and Neck Surgery and Oncology managing HNC in the Nordic countries.

**Results:**

All 21 (100%) Nordic university hospitals responded to the survey. A majority (over 90%) of the patients are discussed at diagnosis in a multidisciplinary tumor board (MDT), but the presence of a palliative care specialist is lacking in 95% of these MDT’s. The patients have access to specialized palliative care units (*n* = 14, 67%), teams (*n* = 10, 48%), and consultants (*n* = 4, 19%) in the majority of the hospitals.

**Conclusion:**

The present results show that specialized palliative care services are available at the Nordic university hospitals. A major finding was that the collaboration between head and neck surgeons, oncologists and palliative care specialists is not well structured and the palliative care pathway of patients with HNC is not systematically organized. We suggest that early integrated palliative care needs to be included as an addition to the already existing HNC care pathways in the Nordic countries.

## Introduction

More than 500,000 patients worldwide are annually diagnosed with Head and Neck squamous cell cancer and this corresponds with 380,000 deaths each year [1]. At the same time various approaches of systemic anti-cancer modalities continue to develop in parallel with innovations in local treatment options [1]. Furthermore, awareness of early signs and symptoms of Head and Neck Cancer (HNC) and efforts to shorten both patient- and health care-related delay have led to improved survival outcome [2]. It is obvious that health care professionals need to implement new strategies for both long-term HNC survivors and patients in palliative care with advanced HNC. This to provide better up to date medical care and psychological support. The literature offers many reports on the physical, psychological, social and emotional needs of patients with HNC [3]. HNC affects many aspects of life that are largely intertwined with social interaction. Eating, talking and facial appearance are factors that can be affected by HNC [4]. Therefore, the emotional distress and psychological impact are not to be underestimated in this patient group [5]. Even cancer survivors may be affected by physical problems and psychological distress affecting the quality of life [6, 7].

Palliative care service aims to optimize the quality of life in patients with advanced and complex diseases such as patients with incurable HNC. Patients with HNC generally experience a high burden of distress, and a holistic approach to meet their needs can be exemplified into a number of domains. It is recommended that medical care and supportive interventions should target the needs of the patients on an individual basis.

The European Healthcare committee (CDSP) issued recommendations regarding palliative care in 2003, and these were updated in 2013 and 2018 [8–10]. The current status of palliative care in Europe was assessed and recommendations for the structure of palliative care organization were discussed. These recommendations have since been updated and discussed further [10]. The World Health Organization (WHO) also recognizes palliative care as fundamental to quality of life for patients suffering from life-threatening illness and urges member states to implement palliative care policies, structure and legislation where appropriate [11].

In 2018 the Lancet Oncology Commission issued a consensus paper on the role of palliative care in the treatment of cancer. Suggestions were made to improve the current status of palliative care in Europe by means of multidisciplinary team efforts and standardized care pathways for all patients [12]. In addition, a policy position paper published in 2017 by the European Society for Medical Oncology (ESMO) advocated for more patient-centred care throughout the whole treatment continuum, from diagnosis to rehabilitation/survivorship or end-of-life care [13]. A multidisciplinary treatment approach was suggested to be integrated in all cancer treatment. Focus should not only be on cancer treatment and survivorship, but also on the integration of other posttreatment aspects. Early-integrated palliative care is strongly promoted to be made available for this patient population.

Furthermore, an evaluation in 2016 by the European Association of Palliative Care (EAPC), considered palliative care in all Nordic countries to be of second highest or highest possible standard of development with Iceland, Norway and Sweden in the top [14]. However, a study from 2018 in Denmark on extended hypofractionated palliative radiotherapy for patients with HNC pointed out the lack of structured palliative regimens within radiotherapy for these patients [15]. Instead, the treatment was shown to be based on local traditions and infrastructure settings at each center. In addition, indications for palliative cancer surgery in this patient group has been under debate for some time [16]. Both quality of life and cost–benefit analyses are not yet fully scrutinized for HNC patients with palliative care and with a short life-expectancy [17, 18].

A Cochrane review from 2017 looked at early palliative treatment for patients with advanced disease [19]. The intent was to compare traditional palliative care with early-integrated palliative care for adult patients with advanced disease. The results suggested that early palliative interventions improved quality of life and symptom burden but had uncertain effects on the incidence of e.g. depression. No major adverse effects were recorded. However, the results need cautious evaluation since only a few, small studies exist within this particular field. Thirty ongoing studies are suggested to increase the certainty behind the results [19].

No worldwide consensus exists on how palliative care should be organized for patients with advanced HNC disease [20]. The aim of this study was to evaluate the current status of the organization of HNC palliative care in the Nordic countries where the treatment of these malignancies is centralized to the university hospitals [21].

## Materials and methods

A questionnaire with both open and multiple-choice questions was designed to identify characteristics of palliative HNC care at the Nordic university hospitals. The items concerned data on the volume of referral area population and number of HNC patients, practices of a multidisciplinary tumor board (MDT) meeting, availability of palliative care professionals, consultants, or teams and access to palliative care units, home team or hospice as well as whether there were guidelines for this domain of HNC care.

The questionnaire was sent to the Departments of Otorhinolaryngology—Head and Neck Surgery (ORL—HNS), and Oncology at all university hospitals in the Nordic countries representing a referral area population of approximately 27 million people (Table 1). The survey was open between May 2018–May 2019 and reminders were sent to non-replying centers. All university hospitals in Denmark (4), Finland (5), Iceland (1), Norway (4), and Sweden (7) responded. All departments of oncology and 20 out of the 21 departments of ORL HNS responded. Oncologists and ORL—Head and Neck surgeons were set to answer specific questions in the questionnaire. Number of new annual HNC patients in each country was obtained from national cancer registries.

**Table 1:** University hospitals (*n* = 21) in the Nordic countries with the size of the referral area population and the annual number of new HNC patients

University hospital	Size of referral area (*n*)	New HNC patients annually (*n*)*
Denmark		
Odense	1,200,000	277
Copenhagen	2,600,000	207
Aarhus	1,300,000	263
Aalborg	600,000	129
Finland		
Oulu	750,000	111
Turku	870,000	121
Tampere	900,000	158
Kuopio	800,000	112
Helsinki	2,200,000	359
Iceland		
Reykjavik	340,000	40
Norway		
Tromsø	480,000	104
Oslo	3,000,000	483
Trondheim	700,000	128
Bergen	1,100,000	178
Sweden		
Örebro^a^	2,110,000	180
Stockholm	2,430,000	300
Linköping	1,070,000	150
Gothenburg	1,900,000	250
Uppsala^a^	2,110,000	75
Umeå	890,000	100
Lund	1,870, 000	270

^*^Data obtained from national cancer registries

^a^The Uppsala-Örebro region has two university hospitals

## Results

### Multidisciplinary tumor board

All of the centers in the Nordic countries have an MDT meeting. All centers, with the exception of one, answered that over 90% of their patients are discussed at an MDT meeting. The majority (20/21, 95%) of the centers answered that no palliative care specialist is present at their MDT meeting.

### Referral, follow-up and guidelines

Thirteen (13/21, 62%) centers had no or little knowledge about how many patients are being referred to specialized palliative care (Table 2). Follow-up of patients with no curative treatment options is performed to an equal extent by oncologists and ORL—Head and Neck surgeons in the Nordic countries. One-fourth of the ORL—Head and Neck surgeons answered that they follow these patients by themselves, 60% occasionally and 15% do not see these patients during follow-up. One-third of the oncologists responded seeing these patients often, 53% occasionally and 14% do not see these patients during follow-up. The majority of the centers (15/21, 72%) were aware of their national guidelines for palliative care. Three (14%) centers have local guidelines, and three (14%) have no guidelines for palliative care (Table 2).

**Table 2:** Responds regarding organization of palliative care

	*n* (%)
Is there a website for palliative care at your center?	
Yes	9 (43)
No	12 (57)
Is advanced surgery used in palliative care at your center (number of responders = 20)	
Often	2 (10)
Occasionally	12 (60)
No	6 (30)
In your opinion, is the palliative care well-organized at your center?	
Yes	19 (90.5)
No	2 (9.5)
Are there guidelines for palliative care at your center?	
National	15 (72)
Local	3 (14)
No	3 (14)
Is there a palliative care specialist present at MDT at your center?	
Yes	1 (5)
No	20 (95)
Are you aware of a proximate number of HNC patients referred to specialized palliative care at your center	
Yes	8 (38)
No	13 (62)

### Palliative care services

Different options for palliative care are available for the patients with HNC in the Nordic countries. Several of the centers answered that they have access to specialized palliative care units (*n* = 14; 67%), teams (*n* = 10; 48%), and consultants (*n* = 4; 19%) (Table 3). Eight (38%) centers answered that they are responsible themselves for the palliative care at their center (Table 3). Specialized palliative care units at university and non-university hospital level, specialized palliative homes care teams, and hospice facilities are available resources for these patients. Thus, the availability of these resources varies among the centers (Table 3). Advanced surgery with palliative intent is performed in the Nordic countries. Two (10%) centers answered that this is performed often, 12 (60%) centers occasionally and six (30%) do not offer this possibility.

**Table 3:** Organization of palliative care for HNC patients at 21 Nordic university hospitals

	(*n*)	%
What palliative care options do you have access to for these patients? (multiple replies possible)		
Specialized palliative care unit at a university hospital	15	71
Specialized palliative care unit at a non-university hospital	14	67
Specialized palliative home care team	21	100
Hospice	16	76
Other	1	5
Who is responsible for palliative care at your center? (multiple replies possible)		
We have a specialized palliative care unit	14	67
We have a specialized palliative care team	10	48
We have a specialized palliative care consultant	4	19
An oncologist at our center takes responsibility	3	14
My team and/or myself	8	38
No one in particular	0	0
Other	1	5

### Organization of palliative care

Most centers answered that their palliative care is well organized and functioning. There was no difference in the opinions between the oncologists and ORL—Head and Neck surgeons. Only two (9,5%) centers regarded their palliative care as not well organized. Nine (43%) centers have a website describing the arrangement of palliative care.

Suggestions for improvement of the palliative care at these centers included better collaboration with primary healthcare and other sectors of society, standardized care pathways, and better access to specialized palliative home care. To develop the collaboration between primary care and hospital care, primary care givers (e.g. general practitioners, nurses) were suggested to take part in the MDT meetings. Further, increasing the available economic resources was suggested as a means to improve the quality of palliative care.

## Discussion

A web-based survey was applied to investigate the status of the organization of palliative HNC care at all university hospitals in the five Nordic countries. The results demonstrate a limited structure for HNC palliative care at these centers. In the majority of the university hospitals specialized palliative care services are available for patients with advanced HNC (Table 3). However, the lack of a structured care pathway was evident at many centers. Further, there seems to be an insufficient collaboration between the treating specialists and the specialized palliative care teams (Table 2).

The number of studies on HNC palliative care and the general awareness of the existing recommendations are rather limited [22]. A recent report analysed the practices and outcomes in Scotland in a series of 84 patients with HNC who had been recommended treatment with palliative intent at the multidisciplinary tumor board (MDT) meeting [23]. The study was the first to specifically look at a systematic palliative intervention and survival in patients with HNC in a real-life setting. The data showed that 21.5% of the patients discussed at the MDT were primarily offered palliative care. Their mean survival time was 151 days and survival up to 18 months was recorded. The long-term survival for patients with incurable advanced disease emphasizes the importance of a well-organized and structured palliative care for this patient group [24].

In a recent population-based study in Sweden approximately 9% of patients were considered for palliative care at the time diagnosis of HNC [25], which is less than reported in the study by Begbie et al. [23].

Palliative care has been redefined not only as end-of-life care but instead, it aims at being a more integrated part of all oncological treatment, and thus collaboration between the treating teams is essential [12]. International professional organizations such as WHO, ESMO, OECD and ESTRO have already identified the need for structured integration of palliative care in national cancer programs [11–13]. The need for an increased knowledge in this field is emphasized.

Even though health care is rather homogenous in the Nordic countries, especially the access to care, the university hospitals are independent units and differences exist across HNC centers within a country and between countries. Structured national palliative care pathways for patients with HNC in the Nordic countries are mainly non-existing at the moment. The goal of equal cancer treatment to all patients could be more easily met and monitored with standardized palliative care pathways for this patient population. This could also contribute to improved research possibilities and evaluation of quality assurance aspects. In the UK, official guidelines for a structured care of HNC patients in palliative care were adapted in 2016 [26]. These guidelines include specific recommendations and care pathways. The existing national guidelines for the management of patients in palliative care with HNC in the Nordic countries offer a suitable platform where such recommendations can be included. In addition, there is an on-going centralization of medical care in several Nordic countries run by governments with the aim to offer a health care system on equal terms.

Selective subgroups of patients with HNC have a relatively short life expectancy and a heavy symptom burden of their disease and, therefore, treatment with the risk of long-term hospitalization, increased morbidity, or even mortality, should be avoided [20, 21, 23, 25]. Thus, the role of a specialized palliative care team is essential in the expanded HNC managing organization. It could be suggested that a palliative team or specialist should be present at each MDT when needed to enhance competence within pain relief, anxiety, psychosocial distress as well as contact with community care. In the occasion of these patients needing extensive palliative care interventions, the care is already initiated and can easily be intensified. Palliative care teams collaborate more frequently with community palliative care providers to guarantee specialized home care and later also end-of-life care. This will further improve communication between hospital care and home care.

## Conclusion

The model of a structured palliative HNC care consists of an organized collaboration between different oncological treatment units or groups. Surgeons, oncologists and palliative care specialists should all collaborate to facilitate individualized treatment for each patient in a palliative situation. This study points out that some of the desired collaboration is currently lacking in the Nordic countries. We suggest that palliative care specialists and/or teams could be integrated into the MDT meetings since early integrated palliative care is to be considered for these patients. The aim should be to establish national standardized palliative care pathways for patients with HNC. These would ideally include the consideration of early integrated palliative care and a systematic referral of all patients with advanced persistent HNC to specialized palliative care teams. Integration of palliative care in the current standardized care pathways for HNC could be recommended to be applied in all Nordic countries.

## References

[1] Szturz P, Vermorken JB (2019). Management of recurrent and metastatic oral cavity cancer: raising the bar a step higher. Oral Oncol.

[2] Mroueh R, Haapaniemi A, Grenman R, Laranne J, Pukkila M, Almangush A, Salo T, Makitie A (2017). Improved outcomes with oral tongue squamous cell carcinoma in Finland. Head Neck.

[3] Isaksson J, Salander P, Lilliehorn S, Laurell G (2016). Living an everyday life with head and neck cancer 2–2.5 years post-diagnosis—a qualitative prospective study of 56 patients. Soc Sci Med.

[4] Lee YH, Goo-Yoshino S, Lew HL, Chi WC, Yen CF, Liao HF, Chen SC, Liou TH (2020). Social participation in head and neck cancer survivors with swallowing disorder: World Health Organization Disability Assessment Schedule 2.0 study. Head Neck.

[5] Windon MJ, D'Souza G, Faraji F, Troy T, Koch WM, Gourin CG, Kiess AP, Pitman KT, Eisele DW, Fakhry C (2019). Priorities, concerns, and regret among patients with head and neck cancer. Cancer.

[6] Miller MC, Shuman AG (2016). Survivorship in head and neck cancer: a primer. JAMA Otolaryngol Head Neck Surg.

[7] Simcock R, Simo R (2016). Follow-up and survivorship in head and neck cancer. Clin Oncol (R Coll Radiol).

[8] (CDSP) EHC (2003) Recommendation Rec (2003) of the Committee of Ministers to member states on the organisation of palliative care Explanatory Memorandum. https://rm.coe.int/09000016809106a0. Accessed 18 Dec 2019

[9] Europe Co (2003) Recommendation Rec (2003) 24 of the Committee of Ministers to member states on the organization of palliative care. Adopted by the Committee of Ministers on 12 November 2003 at the 860th meeting of the Ministers’ Deputies. https://www.coe.int/t/dg3/health/Source/Rec(2003)24_en.pdf. Accessed 31 Aug 2019

[10] Europe Co (2018) The provision of palliative care in Europe. Parliamentary Assembly. Council of Europe. Resolution 2249 (2018). https://assembly.coe.int/nw/xml/XRef/Xref-XML2HTML-en.asp?fileid=25214&lang=en. Accessed 30 Mar 2019

[11] World Health Assembly (2014) Strengthening of palliative care as a component of comprehensive care throughout the life coursee: Report by the Secretariat. https://apps.who.int/iris/handle/10665/158962.

[12] Kaasa S, Loge JH, Aapro M, Albreht T, Anderson R, Bruera E, Brunelli C, Caraceni A, Cervantes A, Currow DC, Deliens L, Fallon M, Gómez-Batiste X, Grotmol KS, Hannon B, Haugen DF, Higginson IJ, Hjermstad MJ, Hui D, Jordan K, Kurita GP, Larkin PJ, Miccinesi G, Nauck F, Pribakovic R, Rodin G, Sjøgren P, Stone P, Zimmermann C, Lundeby T (2018). Integration of oncology and palliative care: a Lancet Oncology Commission. Lancet Oncol.

[13] Jordan K, Aapro M, Kaasa S, Ripamonti CI, Scotté F, Strasser F, Young A, Bruera E, Herrstedt J, Keefe D, Laird B, Walsh D, Douillard JY, Cervantes A (2018). European Society for Medical Oncology (ESMO) position paper on supportive and palliative care. Ann Oncol.

[14] Care EEAoP (2016) Mapping palliative care systems in long term care facilities in Europe https://www.eapcnet.eu/Portals/0/adam/Content/xwkGGSw2ykCLpHNMPZRxkA/Text/WP1_EAPC%2520report%2520Feb_25_2016.pdf Accessed 18 Dec 2019

[15] Laursen M, Specht L, Kristensen CA, Gothelf A, Bernsdorf M, Vogelius I, Friborg J (2018). An extended hypofractionated palliative radiotherapy regimen for head and neck carcinomas. Front Oncol.

[16] Miglani A, Patel VM, Stern CS, Weichman KE, Haigentz M, Ow TJ, Garfein ES (2016). Palliative reconstruction for the management of incurable head and neck cancer. J Reconstr Microsurg.

[17] Rankin T, Mailey B, Suliman A, Dobke M (2015). Palliative reconstructive surgery may improve quality of life in high functioning noncurable head and neck oncologic patients. Ann Plast Surg.

[18] Weber F, Schuss U, Sittel C (2020). Possibilities and limitations of palliative surgery in head and neck cancer patients. HNO.

[19] Haun MW, Estel S, Rücker G, Friederich H-C, Villalobos M, Thomas M, Hartmann M (2017). Early palliative care for adults with advanced cancer. Cochrane Database Syst Rev.

[20] Schenker Y, Arnold RM, Bauman JE, Heron DE, Johnson JT (2016). An enhanced role for palliative care in the multidisciplinary approach to high-risk head and neck cancer. Cancer.

[21] Mäkitie AA, Cange HH, Hammarstedt-Nordenvall L, Gudjonsson A, Jóhannsson J, Laranne J, Mäenpää H, Rikardsen O, Bratland Å, Wessel I, Johansen J, Grau C (2017). Head and neck cancer management in the Nordic countries: an effort to harmonize treatment. Eur Arch Otorhinolaryngol.

[22] Adjei Boakye E, Mohammed KA, Osazuwa-Peters N, Lee MJ, Slomer L, Emuze D, Jenkins WD (2019). Palliative care knowledge, information sources, and beliefs: results of a national survey of adults in the USA. Palliat Support Care.

[23] Begbie FD, Douglas CM, Finlay F, Montgomery J (2019). Palliative intent treatment for head and neck cancer: an analysis of practice and outcomes. J Laryngol Otol.

[24] McCammon SD (2019). Concurrent palliative care in the surgical management of head and neck cancer. J Surg Oncol.

[25] Talani C, Mäkitie A, Beran M, Holmberg E, Laurell G, Farnebo L (2019). Early mortality after diagnosis of cancer of the head and neck—a population-based nationwide study. PLoS ONE.

[26] Cocks H, Ah-See K, Capel M, Taylor P (2016). Palliative and supportive care in head and neck cancer: United Kingdom National Multidisciplinary Guidelines. J Laryngol Otol.

